# Intra-Articular Injection of Hydrolyzed Collagen to Treat Symptoms of Knee Osteoarthritis. A Functional In Vitro Investigation and a Pilot Retrospective Clinical Study

**DOI:** 10.3390/jcm8070975

**Published:** 2019-07-04

**Authors:** Paola De Luca, Alessandra Colombini, Giulia Carimati, Michelangelo Beggio, Laura de Girolamo, Piero Volpi

**Affiliations:** 1 IRCCS Istituto Ortopedico Galeazzi, Via R. Galeazzi 4, 20161 Milano, Italy; 2 Istituto Clinico Humanitas, Via Alessandro Manzoni, 56, 20089 Rozzano Milano, Italy; 3 Policlinico San Marco, Via Francesco Zanotto, 40, 30173 Mestre, Italy

**Keywords:** knee, osteoarthritis, hydrolyzed collagen, intra-articular injection, non-pharmacological therapy

## Abstract

Among all joints affected, knee osteoarthritis has a prevalence of about 10% in men and 13% in women over 60 years old. Knee osteoarthritis has high economic and social costs and may have a devastating impact on patient quality of life. Treatment of symptomatic knee Osteoarthritis may involve oral or topical administration of non-steroidal anti-inflammatory drugs or intra-articular injection of corticosteroids. Recently, a novel injectable collagen formulation (ChondroGrid) consisting of bovine hydrolyzed <3 kDa type I collagen has been developed and is currently available on the market as an injectable medical device. The primary objective of this study was to investigate the in vitro and in vivo effects of ChondroGrid in treating knee osteoarthritis symptoms to assess its safety and performance. Viability and proliferation of ChondroGrid-exposed human chondrocytes derived from five donors were assessed through the Alamar Blue/CyQuant assays. Their expression of *MMP1*/*MMP3* and *TIMP1*/*TIMP3* was then assessed through RT-PCR and that of TGFβ1, IGF-I, and VEGF using ELISA assays. Shape and ECM deposition were assessed using the Bern score after a 28-day ChondroGrid exposure, and collagen deposition was assessed using immunostaining. Records of 20 patients affected by Kellgren Lawrence grade 1 to 4 knee osteoarthritis who received three 4 mg/2 mL ChondroGrid injections 2 weeks apart were then retrospectively assessed to compare VAS, Lequesne, and WOMAC scores collected before and 15, 45, and 225 days after the first injection. ChondroGrid had no effects on the markers under consideration, but induced type-II and inhibited type-I collagen deposition; the Bern score was higher when cells were cultured with ChondroGrid. Patients experienced a 44% Lequesne score and a 55% VAS at moving score reduction. All other scores decreased >70%. ChondroGrid may prompt chondrocytes to produce hyaline cartilage, prevent fibrous tissue formation, and be a safe and effective adjuvant to treat symptomatic knee osteoarthritis.

## 1. Introduction

Osteoarthritis (OA) is the most common musculoskeletal disorder [1] affecting both small and large diarthrodial joints with the hand, hip, and knee being the most affected areas [2]. OA is a disease predominantly affecting the aging population, and the number of those affected is expected to increase in the coming decades [3]. Among all joints affected, knee OA has a prevalence of about 10% in men and 13% in women over 60 years old [4]. Knee OA has high economic and social costs and may have a devastating impact on patient quality of life (QoL) [5,6]. Treatment of symptomatic knee OA may involve oral or topical administration of non-steroidal anti-inflammatory drugs (NSAIDs) or intra-articular injection of corticosteroids. Topical application of NSAIDs calls for multiple daily application to show effectiveness, while oral NSAIDs administration and intra-articular corticosteroid injection have a short-term effect and exhibit several side-effects [7,8,9]. This has led to investigations using non-pharmacological interventions alternatively to or in combination with pharmacological treatments [2,10], including manual and physical therapies [11] and viscosupplementation [12,13]. OA-affected joints exhibit a complex range of structural, tissue, cellular, and biochemical changes. Inflammation mediators, such as IL-1α, IL-1β, TNF-β, and IL-6, are expressed and they, in turn, activate cartilage-degrading enzymes, such as matrix metalloproteinases (MMPs) and a disintegrin and a metalloproteinase with thrombospondin motifs (ADAMTS) [1,14]. The activity of these enzymes leads to the progressive degradation of the extracellular matrix (ECM), including collagen [15]. This observation has led to investigating if exogenous administration of collagen may be beneficial to compensate for this OA-related event. When animal or human synovial and cartilage cells were exposed to different collagen formulations, having a different degree of hydrolyzation or polymerization, they increased the production of hyaluronic acid, while decreasing the release of some inflammation mediators [16,17,18]. Intra-articular injections of a Gly-X-Y collagenic tripeptide in an animal model of osteoarthritis significantly reduced the degradation of the articular cartilage and increased the number of chondrocytes that tested positively to the synthesis of type II collagen [19]. Clinical investigations on the safety and performance of hydrolyzed or polymerized collagen are still few, limited to two randomized clinical trials on a few patients. The first, by Furuzawa-Carballeda et al. [20,21], showed a statistically significant improvement on VAS, WOMAC, and Lequesne indexes after 12 bi-weekly intra-articular injections of 2 mL (13.8 mg) pepsin-treated porcine polymerized, type I collagen versus as many placebo injections. More recently, a double-blind randomized controlled clinical trial on 29 patients [22] showed no significant differences in VAS and Lequesne scores at 3 or 6 months after treatment, in patients that were administered five intra-articular injections of 4 mL (concentration unknown) 300 kDa type I hydrolyzed porcine collagen at 1-week intervals versus patients that were given as many injections of 2.5 mL (25 mg) sodium hyaluronate. Recently, a novel injectable collagen formulation (ChondroGrid (CG), Bioteck, Arcugnano, Italy) consisting of bovine hydrolyzed <3 kDa type I collagen (4 mg/2 mL) has been developed and is currently available on the market as an injectable medical device. The intra-articular treatment consists of three injections: the first two performed 15 days apart, followed by the third, administered one month after the second injection. At present, no investigations have been published concerning CG, neither involving in vitro or in vivo models nor in the clinical settings, and no assumptions can be made on its safety and performance based on previous studies on other intra-articular injectable formulations, given the different dose, collagen molecular weight, and treatment protocol involved. The primary aim of this study was to assess CG safety and effectiveness in vitro by studying human chondrocyte viability when exposed to different doses of CG, and the effect of CG on the expression of different metabolic, angiogenic and trophic markers on an in vitro model of OA. The secondary purpose was to gain preliminary clinical information concerning CG safety and performance in reducing symptoms of knee OA through a pilot retrospective collection and analysis of clinical data.

## 2. Experimental Section

### 2.1. In Vitro Study

#### 2.1.1. Isolation and Expansion of Human Articular Chondrocytes

The study was approved by the Institutional Review Board (M-SPER-015, for use of waste biological material), and specimens were collected under patient informed consent.

Human articular chondrocytes were isolated by enzymatic digestion of articular cartilage portions obtained during total hip arthroplasty from five donors (1 male and 4 women) with a mean age of 50.6 ± 5.8, affected by Kellgren Lawrence grade IV osteoarthritis. The cartilage was harvested with a scalpel from non-weight bearing superficial areas of femoral head, as previously reported [23]; subsequently, the cartilage was cut in small pieces and digested with 0.15% (w/v) Type II Collagenase (Worthington Biochemical Corporation, Lakewood, NJ, USA), for 22 hours at 37 °C under stirring.

The chondrocytes were seeded at a density of 5000 cells/cm^2^ in High Glucose (HG) Dulbecco’s modified Eagle’s medium (DMEM) supplemented with 10% (v/v) FBS, 1% (v/v) 1M HEPES, 1% (v/v) 100 mM Sodium Pyruvate, 1% (v/v) 200 mM L-Glutamine, 1% 10.000 U/mL Penicillin, 10 mg/mL Streptomycin and 1% (v/v) 250 µg/mL Amphotericin B (namely complete medium) and incubated at 37 °C (all reagents from Thermofisher Scientific, Waltham, MA, USA).

#### 2.1.2. IL-1β Treatment Protocol

To simulate an inflammatory environment, after 48 hours in culture in complete medium, the chondrocytes were treated for 48 hours with 1 ng/mL of IL-1β [14,24].

#### 2.1.3. CG Treatment

CG powder was dissolved in DMEM complete medium immediately before the treatment. The cytotoxicity was evaluated assessing the effect of different doses of CG (0.5, 0.75, 1, and 1.5 mg/mL) on the cells (5000/cm^2^) for three and six days, changing the medium at day 3 and repeating the CG treatment. For gene expression analysis and cytokine evaluation (ELISA), the cells were treated for 48 hours with 1 mg/mL of CG in the presence or absence of 1 ng/mL of IL-1β. For histological evaluation, the pellets were cultured with or without chondrogenic medium, in the presence or absence of 1 mg/mL of CG.

#### 2.1.4. Chondrogenic Differentiation in Pellet Culture 

After three passages in culture, 4x10^5^ chondrocytes were centrifuged (2 minutes at 232 *g*) and maintained for 28 days in four different conditions: (1) complete medium (NT); (2) serum-free complete medium supplemented with 1.25 mg/mL human serum albumin, 1 % ITS+1 (1 mg/mL insulin from bovine pancreas, 0.55 mg/mL human transferrin, 0.5 μg/ mL sodium selenite, 50 mg/mL bovine serum albumin, and 470 μg/mL linoleic acid), 0.1 µM dexamethasone, 0.1 mM L-ascorbic acid-2-phosphate, and 10 ng/mL (all from Sigma-Aldrich s.r.l., St. Louis, MO, USA), TGFβ1 (Peprotech, Rocky Hill, NJ, USA), namely chondrogenic medium (C); (3) complete medium supplemented with CG (NT+CG); (4) chondrogenic medium supplemented with CG (C+CG).

#### 2.1.5. Viability Assay (Alamar Blue)

Alamar Blue reagent (Invitrogen, Carlsbad, CA, USA) was used to evaluate the cytotoxicity of different CG concentrations. The cells were seeded in 96-well plates (15000/cm^2^). After 24 hours, the medium was removed from each well and replaced with fresh FBS-free medium, containing 10% v/v Alamar Blue. Plates were incubated for 4 hours at 37 °C, and then the medium emission was measured at 580 nm (excitation 540 nm) using a Victor X3 Plate Reader (Perkin Elmer, Waltham, MA, USA).

#### 2.1.6. Proliferation Assay (CyQuant)

Cell proliferation was assessed using the CyQUANT^®^ cell proliferation assay kit (Invitrogen, Carlsbad, CA, USA) on the same cells used for viability assay. After the Alamar blue assay, the wells were washed twice with PBS and then frozen at −80 °C. The cells were then thawed, and the CyQUANT^®^ GR dye/cell-lysis buffer was added to each sample well. After incubation for 2 to 5 minutes at room temperature (RT), protected from light, the sample fluorescence was measured (excitation 480 nm, emission 520 nm).

A standard calibration curve was done for both Alamar blue and the CyQUANT^®^ assays using a range of chondrocytes from 2500 to 35000 cells.

#### 2.1.7. Gene Expression Analysis

Total RNA was isolated from cell lysates using the PureLink^®^ RNA Mini Kit (Thermo Fisher Scientific, Waltham, MA, USA) and quantified spectrophotometrically by Nanodrop (Thermo Fisher Scientific, Waltham, MA, USA).

For each sample, 800 ng of cDNA was synthesized employing the iScript cDNA Synthesis Kit (Bio-Rad Laboratories, Hercules, CA, USA). Gene expression was evaluated by real-time PCR (StepOne Plus, Thermo Fisher Scientific, Waltham, MA, USA) and performed using TaqMan^®^ Gene Expression Assays (Thermo Fisher Scientific, Waltham, MA, USA).

The expression of matrix metalloproteases *MMP1*, Hs00899658_m1, *MMP3*, Hs00968305_m1 and of their inhibitors *TIMP1*, Hs00171558_m1 and *TIMP3*, Hs00165949_m1 (Thermo Fisher Scientific Waltham, MA, USA) was analyzed before and after IL-1β treatment.

The normalization was performed using the validated housekeeping gene *TBP*, Hs00427620_m1 (Thermo Fisher Scientific) [25]. Data were expressed according to the ΔCt method.

#### 2.1.8. Determination of TGFβ1, IGF-1, and VEGF 

Levels of soluble TGFβ1, IGF-1, and VEGF were detected after 48 hours of treatment in culture medium of cells untreated, treated with 1 ng/mL of IL-1β, and treated with 1 ng/mL of IL-1β + 1 mg/mL of CG, by commercially available ELISA, according to the manufacturer’s protocols (PeproTech, Rocky Hill, NJ, USA).

#### 2.1.9. Histological Analysis 

Pellets were fixed with 10% neutral buffered formalin (Sigma-Aldrich, Gallarate, Milano, Italy), embedded in paraffin, sectioned at 4 μm, and stained with hematoxylin and eosin to evaluate the cell morphology. For semi-quantitative evaluation of Collagen I and Collagen II, the pellet sections were incubated in Rabbit Monoclonal Anti-Collagen Type I, 1:4000 (ab138492, Abcam, Cambridge, CB4 0FL, UK) and Rabbit Polyclonal Anti-Collagen II, 1:100 (ab34712, Abcam) primary antibodies diluted in PBS-BT buffer (PBS buffer supplemented with 5% w/v bovine serum albumin). Sections were then washed with PBS buffer and incubated for one hour with anti-rabbit IgG, (H+L) raised in goat, biotinylated 1:200 (VC-BA-1000-MM15, Vector Laboratories, Burlingame, CA, USA). The Bern Score visual grading system [26] was used for the assessment of the in vitro cartilaginous differentiation, and immunostained sections were scored for the presence of type I and II collagen using a semi-quantitative scoring system as follows: 0 = absence, 1 = mild, 2 = moderate, 3 = marked [27].

### 2.2. Retrospective Clinical Study

Clinical records were selected among those of patients suffering from knee OA and referred to the Knee Surgery and Sports Traumatology Unit, Humanitas Research Hospital, Milan, Italy and to the Policlinico San Marco Hospital, Mestre, Italy. Patients included in this retrospective study (a) were between 18 and 75 years old; (b) were suffering from Kellgren Lawrence [28] grade 1 to 4 knee OA, and (c) underwent treatment with CG according to its indications for use. Exclusion criteria were (a) any disease that interfered with the assessment of knee symptom and function indexes, such as fibromyalgia, Reiter’s syndrome, rheumatoid arthritis, and any other local or systemic immune-mediated disease; (b) BMI ≥ 30; (c) clinical signs of knee infection and/or skin disorders/issues affecting the target knee; (d) intra-articular injections of corticosteroids, hyaluronate, or other formulations during the 3 months before the CG treatment and during the follow-up; (e) surgery at the target knee during the 6 months prior to the CG treatment and during the follow-up; (f) cancer, HIV, HCV; (g) drug or alcohol abuse. Clinical records analyzed in the study reported complete anamnestic and demographic patient’s data; knee AP weight-bearing X-rays collected before treatment; OA severity grade measured by the KL score; Lequesne indexes [29], WOMAC scores [30], and subjective VAS scores, measured at rest and moving. All these data were collected just before the first injection (baseline/T0), and then at the second (T1) and the third (T2) CG injections, as well as 6 months after the third CG injection (FUP).

All patients provided their informed consent to treatment with CG. No ethical committee approval was sought for this study given its retrospective nature and the use of CG according to its manufacturer’s indications for use.

### 2.3. Treatment

The patients received three 2 mL (4mg) CG injections, the first two 15 days apart, and the third one 30 days after the second. The CG injection was carried out according to a superolateral approach to the patella. After injecting CG, the needle was removed, and a sterile gauze was applied over the injection site.

### 2.4. Statistical Analysis

Data collected in the in vitro part of this study were first checked for normality using Kolmogorov–Smirnov and Shapiro–Wilk tests; as their distribution was found to be normal, comparisons between the study endpoints measured under different cell culture conditions were carried out using one-way ANOVA tests followed by pairwise comparisons using the Bonferroni post-hoc test.

Concerning the in vivo part of this investigation, statistical analysis was carried out as follows. Patients’ demographic and characteristics at baseline were described by means of descriptive statistics. To investigate if treatment with CG caused any change among scores collected at the observation time points, distribution of VAS, Lequesne, and WOMAC total, pain, stiffness, and physical function scores were first checked for normality using the Shapiro–Wilk test and, as the distribution was found to be not normal for all of them, scores for each parameter at all different time points were compared using a non-parametric ANOVA Friedman test, followed by pairwise comparisons using the Wilcoxon signed rank test. 

The results of parametric tests are provided as mean ± standard deviation; the results of non-parametric tests are provided as medians and the corresponding interquartile ranges (IQRs). All statistical tests were regarded as significant if *p* < 0.05. All statistical calculations were performed using standard statistical software programs (R System, Ver. 3.3.2 with RMS libraries, R Core Team, 2017 or GraphPad Prism v5.00; GraphPad software, La Jolla, CA, USA).

## 3. Results

### 3.1. In Vitro Assessment

**Figure 1:**
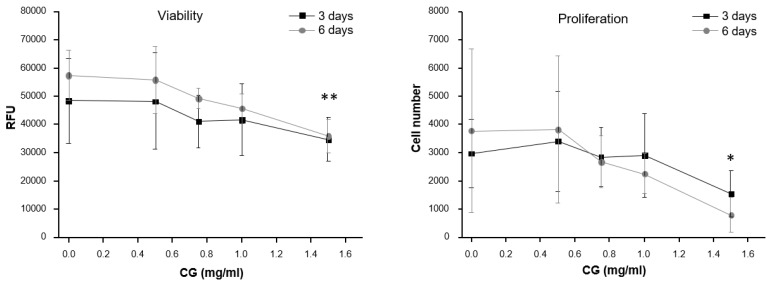
Human chondrocyte viability (left) and proliferation (right) at different CG concentrations. CG 1.5 mg/mL induces a significant decrease in viability (** *p* ≤ 0.001) and proliferation (* *p* ≤ 0.05), with respect to control (no CG) only after 6 days of treatment. No significant differences were observed, at any concentration, between data collected at 3 and 6 days of exposure.

After articular cartilage processing, human chondrocytes obtained [23,31,32] were evaluated for viability and proliferation after 3 and 6 days of exposure to different CG concentrations (Figure 1). A significant difference with control (no CG) was observed only when the higher concentration (1.5 mg/mL) was used for 6 days (*p* < 0.01 for viability and *p* < 0.05 for proliferation). No significant difference was observed between values at 3 and 6 days at any CG concentration. As CG 1.5 mg/mL was observed to affect viability and proliferation, all the following experiments were carried out using CG 1 mg/mL.

**Figure 2:**
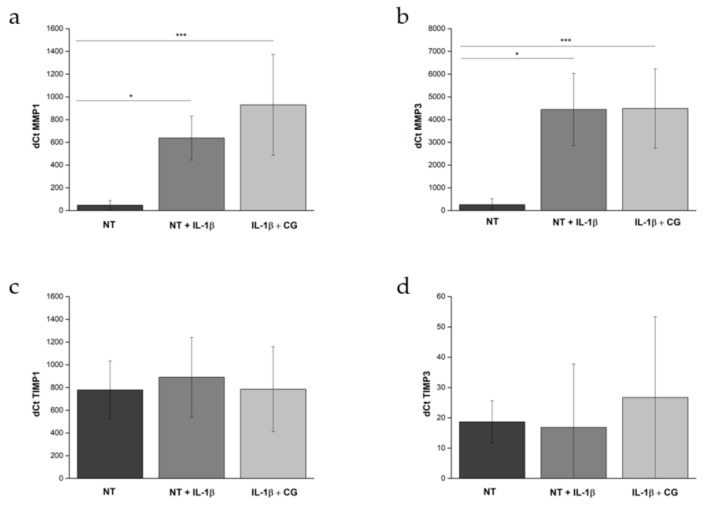
Expression of *MMP1*, *MMP3*, *TIMP1,* and *TIMP3*. An inflammatory response was induced in chondrocytes by exposure to IL-1β. Consequent exposure to CG 1.0 mg/mL (IL-1β + CG) did not induce a significant change in the expression of *MMP1* (**a**), *MMP3* (**b**), *TIMP1* (**c**), *TIMP3* (**d**). NT: control (Not Treated). * *p* ≤ 0.05, *** *p* ≤ 0.001.

CG affected neither the expression of matrix-degrading enzymes (*MMP1* and *MMP3*) nor that of their inhibitors (*TIMP1* and *TIMP3*) (Figure 2).

Moreover, CG did not induce any change in the release of the angiogenic VEGF (676.6 ± 185.7 NT, 1143 ± 233.2 NT + IL-1β and 1147 ± 383.5 IL-1β + CG), or the trophic TGFβ1 and IGF-I (under the detection limits in all samples).

**Figure 3:**
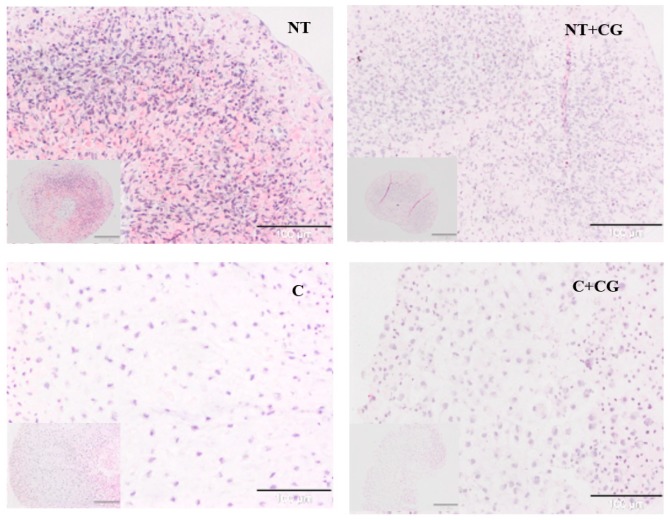
Human chondrocytes cultured under different conditions: NT, not treated; NT+CG, CG 1 mg/mL added; C, chondrogenic medium added; C+CG, chondrogenic medium, and CG 1 mg/mL added. Hematoxylin and eosin staining; bars of small pictures = 100 µm; bars of large pictures = 200 µm.

Irrespective of the culture medium, chondrocytes presented significantly smaller cell density and greater matrix deposition in the presence of CG (Figure 3); the cells kept in the chondrogenic medium and cultured with CG also showed a significantly higher morphology score than controls, having a more rounded shape (Table 1).

**Figure 4:**
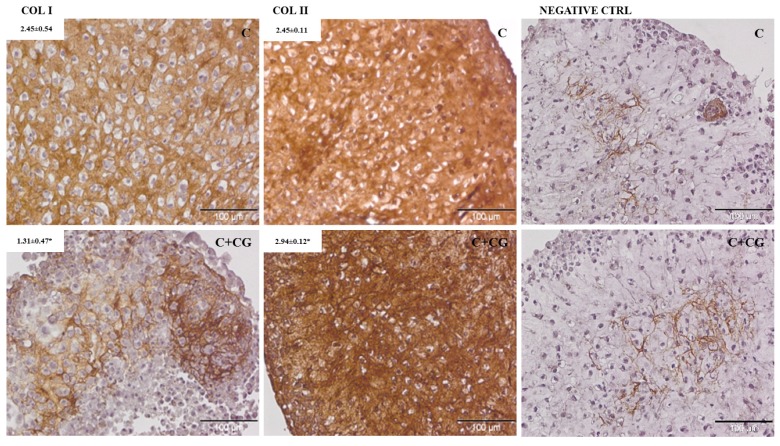
Immunohistochemical analysis of human chondrocytes exposed to a chondrogenic medium (C) or to a chondrogenic medium and CG 1 mg/mL (C + CG). Avidin-biotin detection method. Bar = 100 µm. When chondrocytes were exposed to the chodrogenic medium only, type-I and type-II collagen expression were not significantly different; when they were also exposed to CG 1.0 mg/mL, expression of type-I collagen was inhibited, and that of type-II collagen enhanced. * *p* ≤ 0.05.

When cells were cultured in a chondrogenic medium, they produced both type-I and type-II collagen, whereas when CG was added, high deposition of type-II collagen and inhibition of type-I collagen deposition were observed (Figure 4, Table 2).

### 3.2. Retrospective Clinical Study

The data about 20 non-consecutive patients (14 men and 6 women) with a mean age of 58.1 ± 11.1 (range 35 to 72) were analyzed (Table 3). None of the patients experienced any complications or side effects following CG injections.

**Figure 5:**
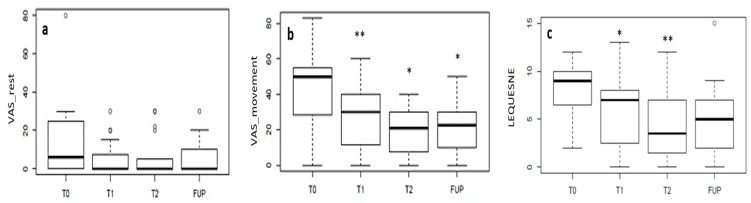
VAS at rest (**a**), when moving (**b**) and Lequesne (**c**) scores. Median VAS at rest did not change significantly after the CG injections. Median VAS when moving decreased significantly after the first injection (T1 vs. baseline, *p* = 0.01) but did not decrease further after the second one (T2); the median Lequesne decreased significantly after the first injection (T1 vs. baseline, *p* = 0.03), did not decrease further after the second injection and was stable at the 6-month follow-up.

Median VAS values at rest did not change significantly with respect to baseline after the CG injections (*p* = 0.1). Median VAS values when moving decreased significantly after the first injection (T1 vs. baseline, *p* = 0.01) but did not decrease further after the second injection (T2 vs. T1, *p* = 0.07), and were stable at the 6-month follow-up (FUP vs. T2, *p* = 0.74) (Figure 5). The median Lequesne index decreased significantly after the first injection (T1 vs. baseline, *p* = 0.01), did not decrease further after the second injection (T2 vs. T1, *p* = 0.06), and was stable at the 6-month follow-up (FUP vs. T2, *p* = 0.58) (Figure 5).

**Figure 6:**
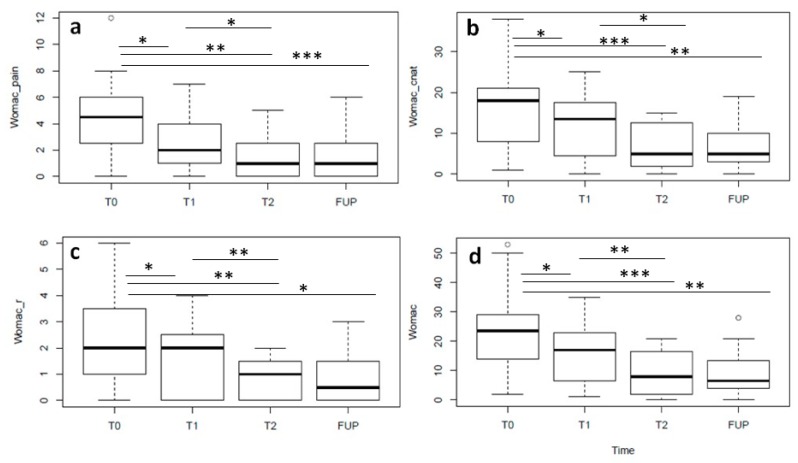
WOMAC pain (**a**), stiffness (**b**), physical function (**c**) sub scores and total WOMAC score (**d**). For all scores, median values at T1 are significantly lower than those at baseline (T1 vs baseline: pain, *p* = 0.03; stiffness, *p* = 0.04; physical function, *p* = 0.04; total score, *p* = 0.03), and median values at T2 are significantly lower than those at T1 (T2 vs T1: pain, p = 0.03; stiffness, *p* = 0.04; physical function, *p* = 0.001; total score, *p* < 0.001). For all scores, median values at the 6-month follow-up were not significantly different than that at T2 and were stable at the 6-month follow-up.

All four WOMAC sub scores median values were significantly lower at T1 than those at baseline (T1 vs baseline: pain, *p* = 0.03; stiffness, *p* = 0.04; physical function, *p* = 0.04; total score, *p* = 0.03), and median values at T2 were significantly lower than those at T1 (T2 vs. T1: pain, *p* = 0.03; stiffness, *p* = 0.04; physical function, *p* = 0.001; total score, *p* < 0.001). For all WOMAC sub scores, median values at the 6-month follow-up were not significantly different from those at T2 (FUP vs. T2: pain, *p* = 0.94; stiffness, *p* = 0.99; physical function, *p* = 0.76; total score, *p* = 0.86) (Figure 6). All the data are summarized in Table 4.

## 4. Discussion

This study has investigated the in vitro and in vivo effects of intra-articular knee injection of ChondroGrid (CG), consisting of bovine collagen hydrolyzed into <3 kDa fragments, to get a preliminary insight on its actual safety and effectiveness profile. The main findings of this study are that CG is a safe product able to provide significant improvements in all the scores at the last follow-up with respect to baseline, probably mediated by the effect of CG on chondrocytes in prompting chondrocytes to produce hyaline cartilage and preventing fibrous tissue formation, as shown by the in vitro study.

In particular, the in vitro results showed that CG negatively affects chondrocyte viability and proliferation at 1.5 mg/mL, a concentration that, given the therapeutic protocol proposed for CG (two performed at 15 days apart and the third at one month after the last injection) and the volume of the synovial fluid in the symptomatic OA knee joint (>10 mL) [33,34], can never be reached in the clinical setting. These results are in contrast with previous observations by Furuzawa et al. [17] who exposed cartilage tissue from five patients with OA for 7 days to 1% (0.6 µg/mL, MW unknown) porcine polymerized-collagen and observed a 3- to 6-fold increase in cell proliferation, but are consistent with those by Nakatani et al. [35] who exposed ATDC5 cells, a murine chondrocyte cell line, to porcine hydrolyzed collagen (1 mg/mL) with an average molecular weight of 5 kDa and did not observe any effect on proliferation. These discrepancies might be ascribed to differences between tissue and cell in vitro cultures and to the concentration as well as the molecular weight of the different formulations; a recent investigation has shown that, in fact, collagen fragments of different lengths may have different effects on cartilage metabolism [36]. In vitro results also demonstrated that CG had no effect on the expression of two of the most common metalloproteinases associated with knee OA, *MMP1,* and *MMP3,* nor on their corresponding inhibitors, *TIMP1* and *TIMP3*. Concerning *TIMP1,* these results are consistent with the observations by Furuzawa et al. [17], who did not observe any *TIMP1* up- or downregulation. This study, therefore, shows that, in the conditions here tested (CH 1 mg/mL; human chondrocytes activated using IL-1β), CG has no metabolic effect on two of the most relevant OA ECM degradation pathways. CG, as expected, also did not affect the production of some well-known trophic (TGFβ1, IGF-I) and proangiogenic (VEGF) factors, indicating this formulation cannot act at a biochemical level to modulate the network of signals involved in the establishment, maintenance, and control of the OA. Interestingly, when chondrocytes were co-cultured with a chondrogenic medium and CG, they better maintained their morphology and phenotype and produced more of the type-II collagen typical of hyaline cartilage, and less type-I collagen, usually found in fibrous cartilage; these results suggest that CG may prompt chondrocytes to produce physiologic articular cartilage, and counterbalance the normal reparative response that would lead, instead, to fibrous tissue formation.

CG was well-tolerated and did not induce significant side-effects. The data showed that the treatment significantly reduced VAS, Lequesne, and WOMAC scores, as also previously reported [17,21,22]. The reduction at 6 months in Lequesne and WOMAC scores were greater than those observed by Furuzawa [17,21] and was achieved using a therapeutic protocol consisting of fewer injections (three instead of five). The decrease in the Lequesne index observed in the present study was also greater than that observed by Martin Martin [22]. Yet, it should be considered that Furuzawa included patients having any degree of knee OA, and Martin Martin included only KL 2 and 3 patients; accordingly, comparison between the results of the present study should be interpreted with caution, and future prospective studies should investigate how these treatments work on homogenous patient subgroups. No comparison can be carried out between the present studies and those cited concerning the VAS scores, as the other authors did not measure VAS during patient movement.

## 5. Conclusions

Results of the present study show that CG may prompt chondrocytes to produce hyaline cartilage and counterbalance the normal reparative response that would lead, instead, to fibrous tissue formation. They also indicate CG may be a safe and effective adjuvant in the treatment of symptomatic knee OA by intra-articular injection. The overall results are extremely promising and highlight the need for further controlled prospective studies to investigate the full extent of the beneficial effects of CG treatment and whether intra-articular CG injection may be more beneficial than other non-pharmacological treatments already available in the clinical practice.

## Figures and Tables

**Table 1:** Bern Scores of human chondrocytes.

Treatment	A Cell Density/Matrix Amount (Score 0–3)	B Cell Morphology (score 0–3)	A+B (Score 0–6)
NT ^a^	0.85 ± 0.76	0.4 ± 0.38	1.25 ± 1
NT + CG ^b^	0.6 ± 0.57	0.75 ± 0.83	1.35 ± 1.28
C ^c^	2.5 ± 0.31 ***§§§	2.10 ± 0.42	4.6 ± 0.63 ***§§§
C+CG ^d^	2.05 ± 0.41 *§§	2.20 ± 0.45 *	4.25 ± 0.77 ***§§§

^a^ NT, not treated; ^b^ NT+CG, CG 1 mg/mL added; ^c^ C, chondrogenic medium; ^d^ C+CG, chondrogenic medium and CG 1 mg/mL; *, vs. NT; §, vs. NT+CG; * or §, *p* < 0.05; ** or §§, *p* < 0.01; *** or §§§, *p* < 0.001.

**Table 2:** Data from immunohistochemical analysis of COL-1 and COL-2 production.

Treatment	Collagen I	Collagen II
C ^a^	2.45 ± 0.54	2.45 ± 0.11
C + CG ^b^	1.31 ± 0.47 *	2.94 ± 0.12 *

^a^ C, chondrogenic medium; ^b^ C+CG, chondrogenic medium and CG 1 mg/mL; * *p* ≤ 0.05.

**Table 3:** Patient characteristics at baseline.

Parameter	Mean ± SD or Y/N (%/%)
Age (years)	58.1 ± 11.1
Weight (kg)	76.5 ± 14.1
Height (cm)	173.7 ± 9
BMI (Kg/m^2^)	25.2 ± 3.3
KL score 1,2,3,4 (%)	4 (20); 11 (55); 3 (15); 3 (10)
M/F (%)	14/6 (70/30)
Diabetes Y/N (%)	2/18 (10/90)
Cardiovascular disorders Y/N (%)	7/13 (35/65)
Metabolic disorders Y/N (%)	4/16 (20/80)
Concomitant treatment Y/N (%)	11/9 (55/45)

**Table 4:** Median values at all time points for the parameters under consideration: VAS at rest and when moving, Lequesne index, WOMAC sub scores concerning pain, stiffness and physical function and total WOMAC score.

	Baseline (Before First Injection)	T1 (15 Days after First Injection)	T2 (30 Days after First Injection)	FUP (About 6 Months after First Injection)	FUP vs. Baseline (%)
Time (days)	N/A	14.5.1 ± 2	22.9 ± 11.2	172.1 ± 22.7	N/A
VAS at rest	6 (22.5)	0 (6.25)	0 (5)	0 (10)	−100%
VAS when moving	50 (23.25)	30 (27.75)	21 (21.25)	22.5 (20)	−55%
Lequesne Index	9 (3.25)	7 (5.25)	3.5 (5.25)	5 (5)	−44%
WOMAC (pain)	4.5 (3.25)	2 (3)	1 (2.25)	1 (2.25)	−77.8%
WOMAC (stiffness)	2 (2.25)	2 (2.25)	1 (2.25)	0.5 (1.25)	−75%
WOMAC (physical function)	18 (13)	13.5 (12.5)	5 (10.25)	5 (7)	−72.2%
WOMAC (Total)	23.5 (14.5)	17 (15.75)	8 (13.75)	6.5 (9.25)	−72.3%

Time is provided as mean ± SD; all other values are provided as median (IQR).
